# Boosting charge separation in organic photovoltaics: unveiling dipole moment variations in excited non-fullerene acceptor layers†

**DOI:** 10.1039/d4sc00917g

**Published:** 2024-07-10

**Authors:** Akira Yamakata, Kosaku Kato, Takumi Urakami, Sota Tsujimura, Kasumi Murayama, Masahiro Higashi, Hirofumi Sato, Yasuhiro Kobori, Tomokazu Umeyama, Hiroshi Imahori

**Affiliations:** a Graduate School of Natural Science and Technology, Okayama University 3-1-1, Tsushima-naka, Kita-ku Okayama 700-8530 Japan yamakata@okayama-u.ac.jp; b Department of Molecular Engineering, Graduate School of Engineering, Kyoto University Nishikyo-ku Kyoto 615-8510 Japan imahori@scl.kyoto-u.ac.jp; c Department of Chemistry, Graduate School of Science, Kobe University 1-1 Rokkodai-cho, Nada-ku Kobe Hyogo 657-8501 Japan; d Department of Complex Systems Science, Graduate School of Informatics, Nagoya University Furo-cho, Chikusa-ku Nagoya 464-8601 Japan higashi@nagoya-u.jp; e Molecular Photoscience Research Center, Kobe University 1-1 Rokkodai-cho, Nada-ku Kobe 657-8501 Japan; f CREST, JST Honcho 4-1-8 Kawaguchi Saitama 332-0012 Japan; g Department of Applied Chemistry, Graduate School of Engineering, University of Hyogo 2167 Shosha Himeji Hyogo 671-2201 Japan; h Institute for Integrated Cell-Material Sciences (WPI-iCeMS), Kyoto University Sakyo-ku Kyoto 606-8501 Japan; i Institute for Liberal Arts and Sciences (ILAS), Kyoto University Kyoto 606-8316 Japan

## Abstract

The power conversion efficiency (PCE) of organic photovoltaics (OPVs) has reached more than 19% due to the rapid development of non-fullerene acceptors (NFAs). To compete with the PCEs (26%) of commercialized silicon-based inorganic photovoltaics, the drawback of OPVs should be minimized. This drawback is the intrinsic large loss of open-circuit voltage; however, a general approach to this issue remains elusive. Here, we report a discovery regarding highly efficient NFAs, specifically ITIC. We found that charge-transfer (CT) and charge dissociation (CD) can occur even in a neat ITIC film without the donor layer. This is surprising, as these processes were previously believed to take place exclusively at donor/acceptor heterojunctions. Femtosecond time-resolved visible to mid-infrared measurements revealed that in the neat ITIC layers, the intermolecular CT immediately proceeds after photoirradiation (<0.1 ps) to form weakly-bound excitons with a binding energy of 0.3 eV, which are further dissociated into free electrons and holes with a time-constant of 56 ps. Theoretical calculations indicate that stacking faults in ITIC (*i.e.*, V-type molecular stacking) induce instantaneous intermolecular CT and CD in the neat ITIC layer. In contrast, J-type stacking does not support such CT and CD. This previously unknown pathway is triggered by the larger dipole moment change on the excited state generated at the lower symmetric V-type molecular stacking of ITIC. This is in sharp contrast with the need of sufficient energy offset for CT and CD at the donor–acceptor heterojunction, leading to the significant voltage loss in conventional OPVs. These results demonstrate that the rational molecular design of NFAs can increase the local dipole moment change on the excited state within the NFA layer. This finding paves the way for a groundbreaking route toward the commercialization of OPVs.

## Abstract 2

Neat ITIC films are found to facilitate charge transfer, dissociation, and free carrier formation without the need for donor layers. This process, driven by V-type molecular stacking, significantly boosts organic photovoltaic efficiency.

## Graphical abstract

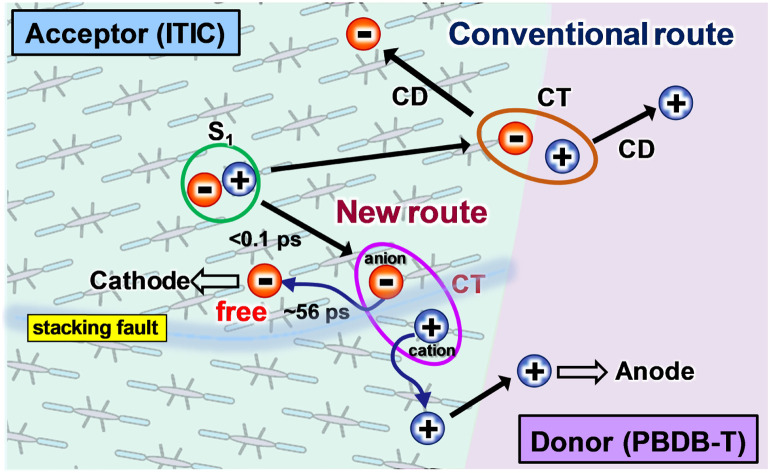

## Introduction

**Scheme 1:**
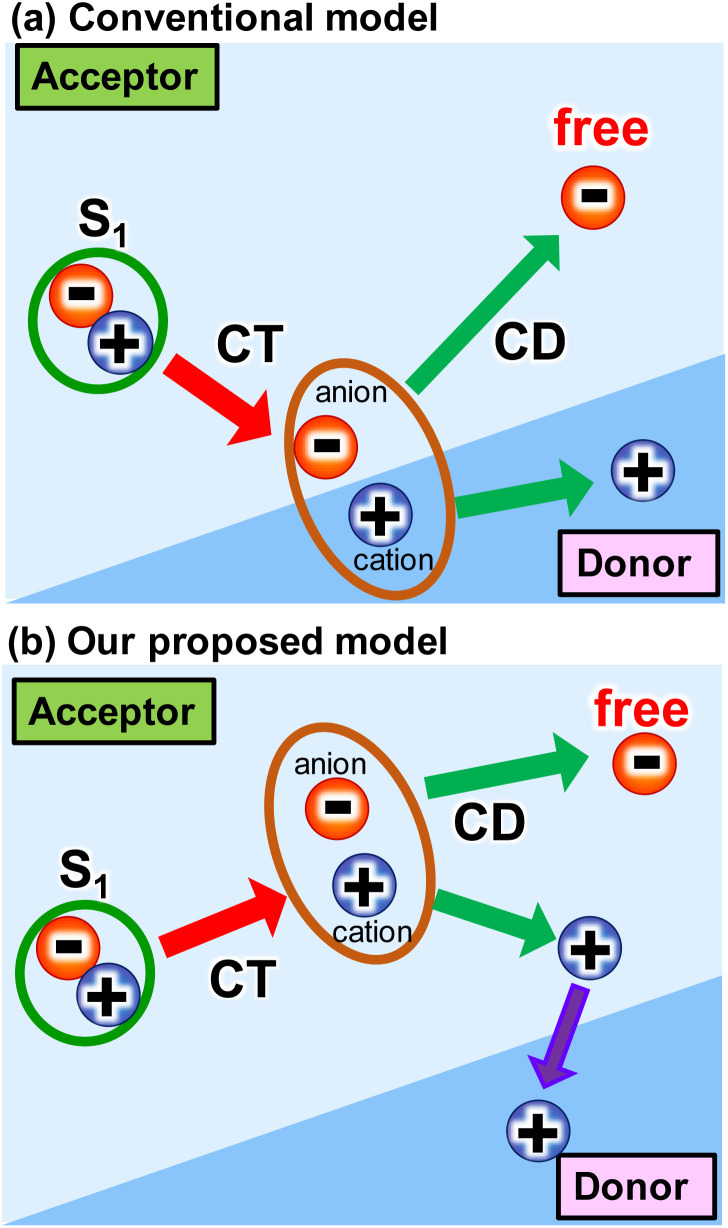
Several intermolecular charge transfer (CT) and charge dissociation (CD) models from singlet (S_1_) state in OPVs.

The use of sustainable and clean energy sources could be a promising option for addressing energy and environmental issues. Photovoltaics have garnered much attention due to their ability to directly convert solar energy to electricity. This is because solar energy is a substantially inexhaustible and clean energy source. In this regard, organic photovoltaics (OPVs) have been intensively studied due to their cost-effectiveness, flexibility, and aesthetical design.^1–7^ Recently, the power conversion efficiency (PCE) of OPVs has exceeded 19% due to the rapid development of non-fullerene acceptors (NFAs).^8,9^ To compete with the PCEs (26%) of commercialized silicon-based inorganic photovoltaics (PVs), the drawbacks of OPVs should be minimized. One of the drawbacks is the intrinsic large loss of open-circuit voltage (*V*_OC_), partly because OPVs require a donor–acceptor heterojunction for charge-transfer (CT) from singlet (S_1_) state and charge dissociation (CD) at the interface.^10^ At the early stage of the development of acceptor molecules (*i.e.*, fullerene derivatives), the energy offset between the S_1_ state and CT state was thought to be more than 0.3 eV for these processes (Scheme 1a). However, the recent emergence of NFAs has shown efficient charge generation with a small energy offset.^10,11^ Recently, Wang *et al.*^12^ reported that charge transfer and dissociation can occur even in a neat Y6 layer using time-resolved visible to near-infrared (NIR) transient absorption (TA) spectroscopy. This charge separation in Y6 was further investigated by studying temperature dependence,^13^ pump-pulse intensity dependence,^14^ and by varying the mixing ratio with PTB7-Th donors.^15^ Photo-Hall and quasi-steady-state photoinduced absorption measurements also support the formation of free carriers in Y6.^16^ Similarly, charge separation was observed in IEICO-4F, IT-4F, and ITIC using the triplet sensitizer PtOEP and inorganic donors such as CuSCN.^17^ Despite these studies, the underlying mechanism of efficient charge generation in NFAs remains unclear. Thus, a general approach to the minimization of the *V*_OC_ loss still remains elusive.^4,18,19^

TA spectroscopy is a powerful tool that enables the observation of photoexcitation dynamics with ultrafast time resolution.^20–23^ There have already been many reports on TA measurements of NFAs.^10,23–27^ However, most of these conventional measurements were performed in the visible and NIR regions (400–1500 nm). These absorptions originate from the electronic transitions between excitonic states such as the S_1_ state to higher S_*n*_ states, hence these measurements provide information about the localized carriers around the molecules. Because the PCE of OPVs depends on CT and CD with the subsequent transport/collection processes, understanding the binding states of carriers is indispensable for increasing the PCEs of OPVs. It is known that the delocalized free carriers exhibit optical response in the mid-infrared (MIR) region (4000–400 cm^−1^) *via* intra-band transition and give structureless broad absorption in the MIR region.^28^ Hence, MIR-TA measurements are often applied to study the behavior of free carriers in inorganic semiconductors such as photocatalysts and dye-sensitized solar cells.^28–31^ In contrast, MIR-TA measurements have not been used for organic materials. Few exceptions include conductive polymers and highly crystalline organic semiconductors^32,33^ where free carriers have been observed for single-crystalline rubrene^34^ and C10-DNTT.^35^ As such, it is still questionable whether free carriers really exist in NFAs films prepared by simple spin-coating and drop-casting. MIR-TA experiments were performed for NFAs to study the change of molecular vibrations, but the existence of free carriers was not confirmed because of the limited spectral ranges.^36,37^ Overall, broadband MIR-TA measurements are prerequisite to monitor the CT and CD processes as well as to identify the localized and delocalized states of photogenerated charge carriers in the NFAs film.

In this study, we found that CD as well as CT proceeds even in a neat ITIC (2,2'-[[6,6,12,12-tetrakis(4-hexylphenyl)-6,12-dihydrodithieno[2,3-*d*:2′,3′-*d*′]-*s*-indaceno[1,2-*b*:5,6-*b*′]dithiophene-2,8-diyl]bis[methylidyne-(3-oxo-1*H*-indene-2,1(3*H*)-diylidene)]]bis[propanedinitrile]) film without a donor layer (Scheme 1b). This was observed using broadband visible to MIR TA measurements, where ITIC is one of the most highly efficient NFAs.^38,39^ After photoexcitation of the neat ITIC film, a broad absorption peak appeared at 3000 cm^−1^ (0.37 eV), indicating the formation of weakly-bound excitons such as an intermolecular CT state with a binding energy of 0.37 eV. This peak disappeared with a time constant of 56 ps, leading to a flat MIR absorption from 1800 to 3800 cm^−1^, indicating complete dissociation of bound excitons and the formation of free carriers. The vibrational frequency shift of C≡N groups verifies the formation of these intermolecular CT, and theoretical calculations predicts that such intermolecular CT and CD can proceed at the stacking fault of the ITIC layers (*i.e.*, V-type molecular stacking). This previously unknown pathway is triggered by the larger dipole moment change on the excited state generated at the lower symmetric V-type molecular stacking of ITIC. This is in sharp contrast with the need of sufficient energy offset for CT and CD at the donor–acceptor heterojunction, leading to the significant voltage loss in conventional OPVs. These results demonstrate that the rational molecular design of NFAs can increase the local dipole moment change on the excited state within in the NFA layer.

## Results and discussion

### Transient absorption of neat ITIC film

**Fig. 1:**
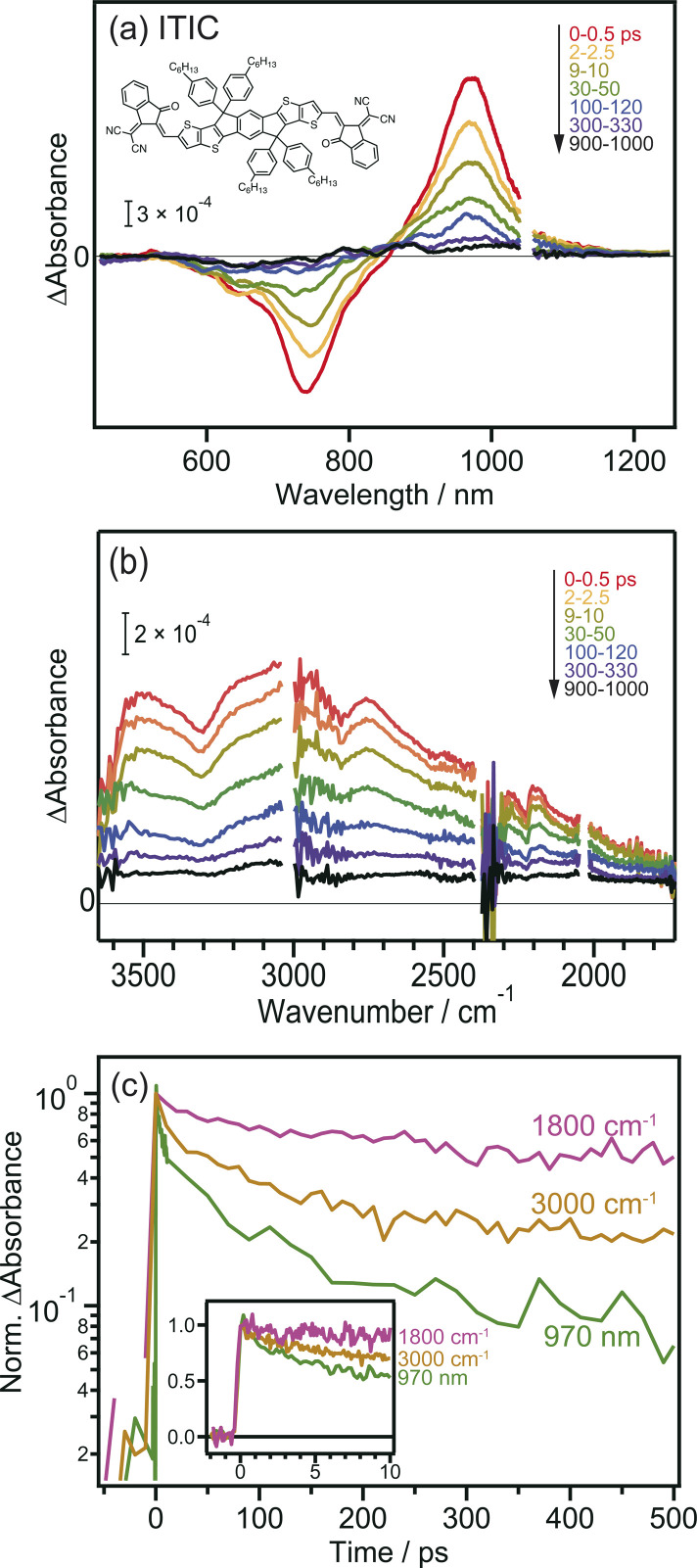
Transient absorption spectra of neat ITIC film after 710 nm laser pulse irradiation. (a) Visible to NIR region. (b) MIR region. (c) Decay kinetics of the transient absorption. The chemical structure of ITIC is also shown in the inset.

Prior to the MIR-TA measurements, conventional VIS-NIR TA measurements were performed for the neat ITIC film. The photoexcitation of the ITIC film by 710 nm laser pulse irradiation caused a negative peak at 740 nm and a positive peak at 970 nm (Fig. 1a). These signals were assigned to the ground-state (S_0_) bleaching and transition of the excited bound state (S_1_) to the higher states (S_*n*_), respectively. The observed TA spectra in the visible region are identical to those reported in previous studies,^40^ and consistent results were obtained in our experiments, showing that the band intensities at 740 and 970 nm simply decrease from 0 to 1000 ps.

In contrast, the TA spectra obtained in the MIR region have unique characteristics. A broad peak appeared around 3000 cm^−1^ immediately after excitation but disappeared within ∼300 ps (Fig. 1b). This peak has been observed in previous reports and is ascribed to the transition of intermolecularly formed bound electron–hole pairs (bound polaron pairs).^36^ The appearance of this peak clearly shows that electrons and holes are separated to neighboring ITIC molecules forming intermolecular CT state, *i.e.*, S_1_ state changed to intermolecular CT state even in the neat ITIC film. The binding energy was estimated to be ∼0.37 eV from the absorption peak at 3000 cm^−1^ (0.37 eV). However, it is noted that the spectral shape changes as the delay time increases. The peak at ∼3000 cm^−1^ disappears within ∼300 ps, and a very flat absorption spanning from 3800 to 1800 cm^−1^ emerges after 300 ps, as will be more clearly shown later by the global analysis (Fig. 3). Such broad TA signals are often observed in inorganic semiconductor photocatalysts such as TiO_2_ (ref. 41) and SrTiO_3_,^42^ and are ascribed to the intraband transition of free carriers,^43^ suggesting the formation of free carriers in ITIC through CD. The signal intensity of these MIR signals observed at 3000 cm^−1^ and 1800 cm^−1^ both linearly increased with increasing laser fluence, as is the case for the peak at 970 nm (Fig. S3†). These results indicate that the observed MIR TA signals are not due to thermal artifacts, but due to the charge carriers generated by the one-photon absorption process in the neat ITIC film, implying that both intermolecular CT and CD proceed in the neat ITIC film. It has been widely believed that intermolecular CT proceeds in the donor–acceptor heterojunctions. However, these results unambiguously corroborate that both intermolecular CT and CD proceed in the neat ITIC film. It should be noted that the decay of TA signals depends on the wavelength of the probe light, where the decay becomes slower as the wavelength increases (Fig. 1c). This indicates that the probe light with longer wavelengths is observing photocarriers with lower binding energies. Consequently, the result suggests that photocarriers with lower binding energies have significantly longer lifetimes. This is consistent with the sequential process where S_1_ states form CT states, and CT states subsequently form free carriers, aligning with the observed time series.

### Transient absorption of PBDB-T:ITIC blend film

**Fig. 2:**
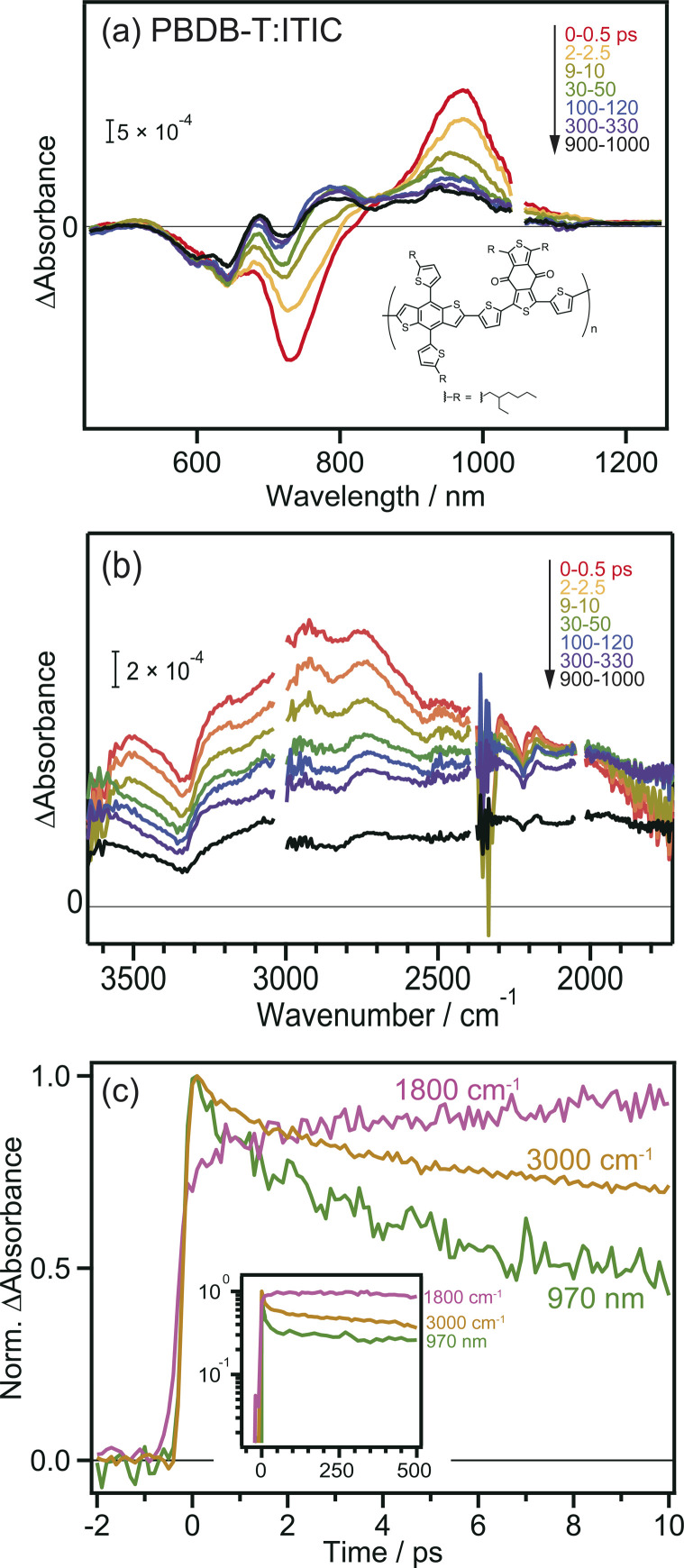
Transient absorption spectra of PBDB-T:ITIC film after 710 nm laser pulse irradiation. (a) Visible to NIR region. (b) MIR region. (c) Decay kinetics of the transient absorption. The chemical structure of PBDB-T is also shown in the inset.

To further confirm the intermolecular CT and CD proceeding in the ITIC layer, the same experiments were performed for the PBDB-T:ITIC blend film, because mixing ITIC with a donor such as PBDB-T would enhance the CT of the bound exciton. In this experiment, ITIC was selectively excited by 710 nm pulses, and TA was measured from the visible to the MIR region. In the visible to NIR region (Fig. 2a), the TA spectra at 0 ps were identical to those of the neat ITIC film (Fig. 1a), where negative and positive peaks were observed at 740 nm and 970 nm, respectively. However, after ∼100 ps, the negative peak shifted from 740 nm to 640 nm, and positive peaks appeared at 690 and 780 nm. This absorption is characteristic of cationic PBDB-T, suggesting that hole transfer proceeds from ITIC to PBDB-T, as reported in a previous study.^40^ However, it is difficult to discuss the details of the CT and CD dynamics only from them because the TAs of various excited states are overlapped in the visible to NIR region. For example, holes in PBDB-T give positive absorption around 930 nm,^40^ but the intensity change at 930 nm does not always reflect the number of holes in PBDB-T because other species such as bound electrons and radical anions of acceptors also give signals at 930 nm. Hence, as shown in Fig. S4a,† it is difficult to estimate the CD timescale from the time profile at 930 nm.

The broad absorption observed in the MIR region for the blend film, as in the case of the neat ITIC film, gradually decreased in intensity to flatten the shape over time (Fig. 2b). This behavior is similar to that of the neat ITIC film (Fig. 1b), but the magnitude of the flat component is 3.7 times larger than that of the neat ITIC film at 1000 ps: the intensity at 1800 cm^−1^ at 1000 ps (∼1.0 × 10^−4^ and ∼4.8 × 10^−4^ for the neat and blend films, respectively) is normalized by the initial peak intensity at 3100 cm^−1^ at 0–0.5 ps (∼1.2 × 10^−3^ and ∼1.6 × 10^−3^ for the neat and blend films, respectively). These results suggest that the formation of donor–acceptor heterojunction enhances the dissociation of the bound exciton, increasing the number of free electrons in ITIC.

The decay kinetics of S_1_, CT state and free electrons observed by the TAs at 1800 cm^−1^, 3000 cm^−1^, and 970 nm provide detailed insights into the CD process at the PBDB-T:ITIC interface. As shown in Fig. 2c, the TA signals at 970 nm and 3000 cm^−1^ monotonically decrease from 0 to 10 ps, while the signal at 1800 cm^−1^ increases (though this increase is not prominent for neat ITIC due to the lower CD efficiency). Furthermore, the signal at 1800 cm^−1^ remains stable for up to 500 ps (inset of Fig. 2c), resulting in a significantly longer lifetime compared to neat ITIC (Fig. S4b†). These results indicate that CD, forming free electrons, occurs within tens of picoseconds and support the idea that the formation of the PBDB-T:ITIC heterojunction can enhance CD and prevent charge recombination. The hole transfer from ITIC to PBDB-T in the PBDB-T blend film was also verified by light-induced time-resolved electron paramagnetic resonance (TREPR). As shown in Fig. S6, S7 and Table S1,† polaron pairs of PBDB-T^+^˙ and ITIC^−^˙ are formed upon photoexcitation, confirming that charge transfer occurs at the heterojunction.

To confirm that the long-lived flat MIR signal observed in Fig. 2c did not originate from the excitation of PBDB-T, TA measurements were performed for the neat PBDB-T film. A broad absorption was also observed at approximately 3100 cm^−1^ (Fig. S5†). However, the peak intensity monotonically decreased with time, and the long-lived flat component was absent. This result suggests that free carriers are not generated in the neat PBDB-T film, or are generated at a much lower level than in the neat ITIC film. This confirms that the long-lived flat mid-IR TA signal does not originate from excitons in PBDB-T but from free electrons generated in the ITIC domain after CD.

### Global analysis of the transient absorption of ITIC and PBDB-T:ITIC film

**Fig. 3:**
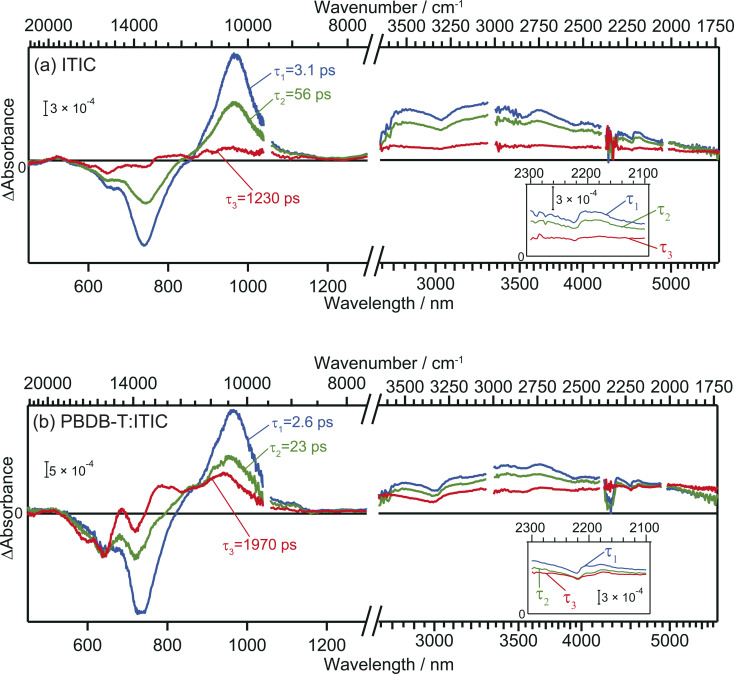
Global analysis of the transient absorption spectra of neat ITIC (a) and PBDB-T:ITIC blend film (b). Inset is the enlarged view around 2200 cm^−1^.

The TA spectra of ITIC were analyzed globally for the visible and mid-IR region. Three components of different lifetimes (*τ*_1_ = 3.1 ps, *τ*_2_ = 56 ps, and *τ*_3_ = 1230 ps) were extracted, as shown in Fig. 3a. Component 1 has negative and positive peaks at 720 nm and 970 nm, respectively, as reported in previous studies.^40^ These bands are assigned to the ground state bleach and the electronic excitation from S_1_ to higher states, respectively. In the MIR region, pump pulse irradiation causes a broad positive peak around 3300 nm (3000 cm^−1^, ∼0.37 eV). This absorption is assigned to the optical dissociation of the bound exciton,^36^ and the binding energy was estimated as ∼0.37 eV. This value is reasonable compared to that estimated by previous studies of 0.2–0.5 eV.^44,45^

The spectral shape of component 2 is similar to component 1 and just the amplitude becomes smaller. This result suggests that the charge carriers in component 2 are in a similar environmental condition as component 1, and indicates that the component 1 simply decays *via* vibration relaxation to form the component 2 without CD.^40,46^ The time constant of this relaxation is estimated to be *τ*_1_ = 3.1 ps.

In contrast, the spectral shape of component 3 was remarkably different from components 1 and 2: the positive and negative peaks at 970 and 740 nm were almost diminished but the relative intensity of negative peak at 640 nm increased. Furthermore, the peak observed at 3000 cm^−1^ disappeared, and a new flat absorption appeared in the entire mid-IR region from 3800 to 1750 cm^−1^. This spectral shape is characteristic of the absorption of free electrons in semiconductors.^28–30^ Therefore, this result indicates that the transition from component 2 to 3 corresponds to CD, resulting in the formation of free electrons. The time constant of this CD was estimated to be *τ*_2_ = 56 ps, and the lifetime of the free carriers is estimated to be *τ*_3_ = 1230 ps. It is worth noting that the negative peak at 640 nm and the flat mid-IR absorption are correlated, suggesting that photoexcitation by the 640 nm pump generates free electrons. The mechanism will be discussed based on DFT calculations later.

In the case of the PBDB-T:ITIC blend film, three components with different lifetimes (*τ*_1_ = 2.6 ps, *τ*_2_ = 23 ps, and *τ*_3_ = 1970 ps) were extracted (Fig. 3b). Component 1 displays negative peaks at 640 nm and 730 nm, along with positive peaks at 970 nm and 3000 cm^−1^. The shape of component 1 is almost identical to that of component 1 of the neat ITIC film. This result is reasonable since the ITIC domain is selectively photoexcited by using a 710 nm laser pulse, confirming that these absorptions are attributed to the photocarriers generated in the ITIC domain.

The spectral shape of component 2 is almost identical to that of component 1. This result is the same as observed for the neat ITIC film, suggesting that the transition of component 1 to component 2 arises from the simple vibrational relaxation of the component 1. The lifetime of component 1 is estimated to be *τ*_1_ = 2.6 ps, which is also identical to that in the neat ITIC within experimental errors.

On the contrary, the spectral shape of component 3 underwent significant changes in comparison to components 1 and 2. The flat absorption of free electrons in ITIC is also observed at 3800–1750 cm^−1^, but the spectral shape in visible region is different, where the negative peak around 640 nm increased further, and new positive peaks emerged at approximately 690 nm and 780 nm. This spectrum differs from component 3 of the neat ITIC film but aligns with that of the cationic PBDB-T.^40^ These results clearly show that holes generated in the ITIC domains are transferred to the PBDB-T domain and free electrons are remained in the ITIC domain. The rate constant of this CD (*τ*_2_ = 23 ps) is faster than that observed in the neat ITIC film (*τ*_2_ = 56 ps), and the signal intensity of free electrons was higher than in the neat ITIC film. Furthermore, the lifetime of this CD state (*τ*_3_ = 1970 ps) is longer than that in the neat ITIC film (*τ*_3_ = 1230 ps). These results support that the CD proceeds more effectively at the heterojunction than in the neat ITIC.

### Analyzing the local charge state of ITIC molecules through CN vibrations

CN groups are added as electron-withdrawing groups of the acceptor moieties in acceptor–donor–acceptor type NFAs where they play essential roles. The frequency shift of CN groups can be used to monitor the CT and CD in the neat ITIC film since vibrational frequencies are highly sensitive to changes in local electron density of molecules.^36,37,47^ DFT calculation predicts that the formation of the S_1_ state induces an internal CT with a 20 cm^−1^ red-shift, and that of the anionic ITIC state *via* intermolecular CT shows further red-shift (Table S2†).

**Fig. 4:**
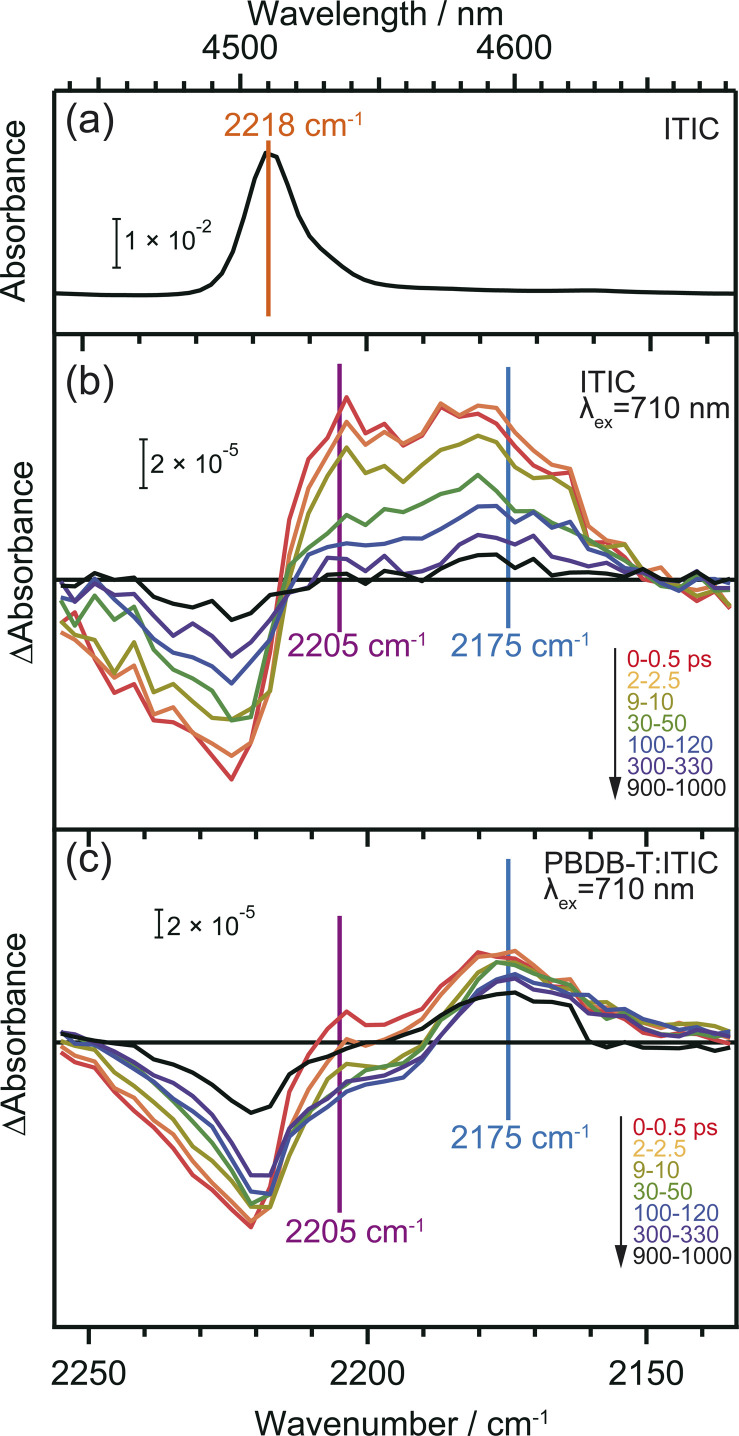
Vibrational spectra of CN groups in ITIC. Steady-state FT-IR spectrum of neat ITIC film (a). Transient IR absorption spectra of neat ITIC film (b) and PBDB-T:ITIC blend film (c) irradiated by 710 nm pump pulse. Broad absorption by photocarriers was subtracted to extract the change of CN vibrations.

Fig. 4 displays the ground state FT-IR and differential transient IR spectra of CN groups in the neat ITIC film. The negative and positive peaks observed at 2224 cm^−1^ and 2205 cm^−1^ after irradiation with a 710 nm pump laser pulse are attributed to ground state bleaching and S_1_ state, respectively. This attribution is based on the fact that S_1_ states cause internal CT, leading to an increase in electron density around the CN group (Table S2†). These two peaks have also been observed in other NFAs such as TT-T-DCl and TT-FT-DCl.^37^ However, the positive peak observed at 2175 cm^−1^ in the neat ITIC film did not appear in either TT-T-DCl or TT-FT-DCl.^37^ Based on DFT calculations, this peak is assigned to the anionic ITIC formed through intermolecular CT. CT also results in the formation of cationic ITIC, causing a blue shift (Table S2†). However, the oscillator strength of the cationic state is much smaller than that of the anionic state, suggesting the possible absence in the TA spectra. Detection of cationic ITIC is difficult even by ns-TREPR (Fig. S8†), as most of the charge carriers recombine within the time resolution of TREPR. However, CT does not always occur in all NFAs since it was absent on TT-T-DCl and TT-FT-DCl.^37^ The power conversion efficiency of PBDB-T:TT-T-DCl and PBDB-T:TT-FT-DCl are 3.41% and 7.13%, respectively, whereas that of PBDB-T:ITIC is 11.3%.^38^ More efficient NFAs tend to induce CT in neat NFA films, since it can prevent geminate recombination.

In the case of the PBDB-T:ITIC blend film (Fig. 4c), the two red-shifted positive peaks were also observed at around 2205 and 2175 cm^−1^ with a negative peak at 2221 cm^−1^. This result suggests that both S_1_ and anionic ITIC states are formed within the ITIC domain. However, it is evident that the peak of S_1_ state diminishes rapidly within 30 ps, while the peak of anionic ITIC persist for approximately 1 ns. These results also support that the CD more effectively proceeds at the donor–acceptor interface. It is noted that the peak width of the negative absorption at ∼2224 cm^−1^ is much wider than the peak width of the ground state CN vibration measured by FT-IR. This result indicates that the ITIC molecules, which induce charge separation, are in more inhomogeneous states.

**Fig. 5:**
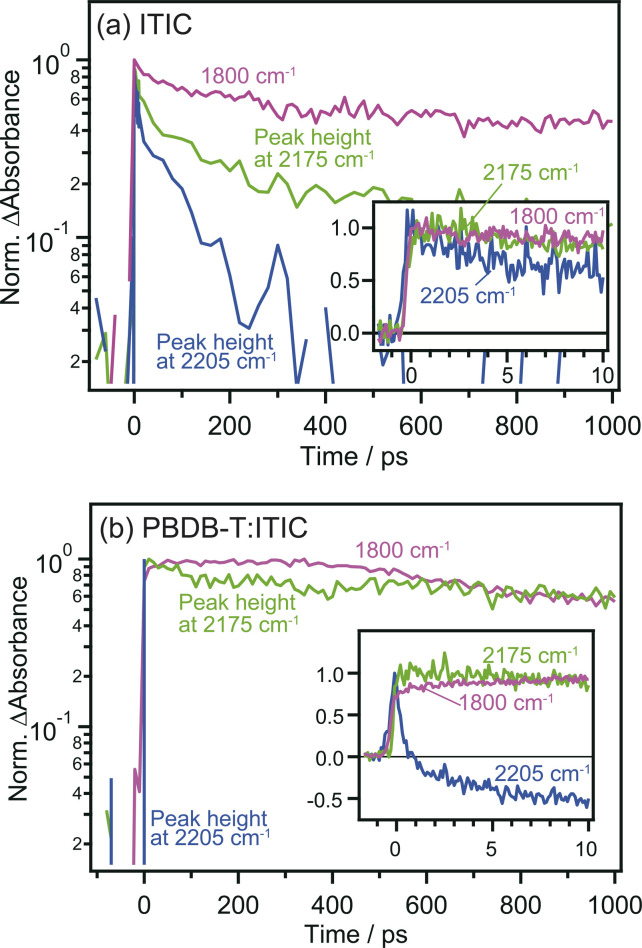
Time profiles of excited S_1_ state, anionic ITIC, and free electrons measured at 2205, 2175, and 1800 cm^−1^ in neat ITIC (a) and PBDB-T:ITIC blend films (b), respectively. The peak heights at 2205 and 2175 cm^−1^ were calculated after subtracting the broad absorptions by photocarriers to extract the change of CN vibrations.

The comparison of the decay curves of S_1_, anionic ITIC, and free electrons measured at 2205, 2175, and 1800 cm^−1^ provides more detailed insights into intermolecular CT and CD dynamics. In the case of the neat ITIC film (Fig. 5a), S_1_ and anionic ITIC spontaneously appear after photoirradiation, indicating that intermolecular CT proceeds within the time-resolution of 90 fs. These signal intensities decrease within hundreds of picoseconds, with lifetimes following the order of free electrons > anionic ITIC > S_1_. This is reasonable since charge recombination becomes slower as the separation distance between electrons and holes increases. On the contrary, in the case of the PBDB-T:ITIC blend film (Fig. 5b), both S_1_ and anionic ITIC spontaneously appear after photoirradiation as is the case of neat ITIC. However, S_1_ decays much faster than that in the neat ITIC. The decay of anionic ITIC and free electrons occurs at a similar rate, especially after 500 ps, and is slow compared to the neat ITIC. These results suggest that free electrons and anionic ITIC are in equilibrium in these time regions.

### Mechanism of charge transfer and charge dissociation in neat ITIC film

We have observed that the intermolecular CT and CD to form free electrons and holes can proceed even in the neat ITIC film without a donor–acceptor interface. The most plausible mechanism for this process lies in the inherent inhomogeneity of the prepared ITIC film. The energy levels of the excitonic states in NFAs can strongly depend on the stacking nature of their π-conjugation systems.^11^ Moreover, the dynamics of S_1_ states within NFA films can be profoundly influenced by molecular interactions within dimers formed in the film.^12,48^ To delve further into the impact of π–π stacking interactions, theoretical calculations were conducted for both the monomer and four representative dimers of ITIC, namely J1, V1, V2, and V2′, as depicted in Fig. S10 and S11.† In principle, ITIC crystals are formed *via* J-type conjugation^49,50^ but V-type π–π interaction is also present at defects such as stacking faults as demonstrated in Fig. S12.† Therefore, these dimers serve as a fundamental model for examining the effects of stacking structures.

**Fig. 6:**
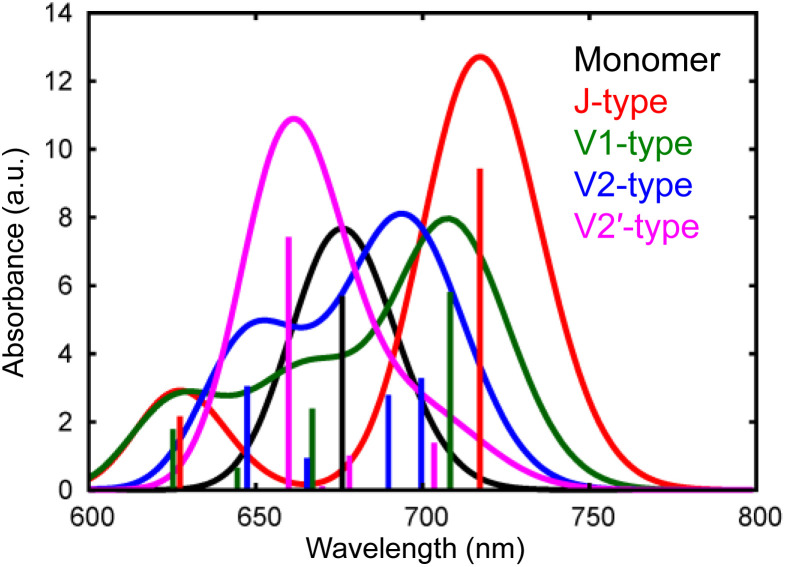
Calculated absorption spectra of different types of ITIC dimers (J, V1, V2, and V2′-type) as well as a monomer.

The simulated photophysical properties, including absorption spectra, peak energies, oscillator strengths, and dipole moment changes, were calculated and are compared, as depicted in Table 1. It is evident from Fig. 6 that the monomer displays a singular absorption peak at 677 nm in the steady-state absorption spectrum, whereas the dimers exhibit several peaks corresponding to the excitations from S_0_ to S_1_, S_2_, S_3_, S_4_, and so forth. Particularly noteworthy is the observation that low-symmetric dimers (V1, V2, and V2′) exhibit changes in dipole moments upon photoexcitation, whereas no changes occur in the higher symmetric J-type dimer (Table 1). It is well known that symmetry breaking can enhance the charge separation in organic medium,^51–55^ hence these results suggest that photoexcitation of these low-symmetric dimers induces the spontaneous intermolecular CT, and Fig. S11† clearly shows that electrons and holes are separated in each molecule. Importantly, the dipole moment tends to increase with higher excitations beyond the S_1_ state. For instance, the transition from S_0_ to S_4_ yields changes of 18.14 D, 10.45 D, and 4.54 D for V1, V2, and V2′ dimers, respectively. In contrast, the changes are 1.50 D, 3.15 D, and 3.26 D from S_0_ to S_1_. These results indicate that absorption of higher-energy photons leads to more efficient intermolecular CT under specific π–π interaction. Actually, the excitation of V-type dimers to S_3_ or S_4_ gives absorption at ∼640 nm, whereas the excitation to S_1_ of J-type dimers gives absorption at 740 nm. Hence, photoexcitation at 640 nm will create more free electrons than at 740 nm. This prediction supports our experimental results that the appearance of free electron signal in the mid-IR region is associated with the ground state bleach signal at 640 nm, rather than at 740 nm (Fig. 3a).

**Table 1:** Calculated results of the absorption energy *E*_abs_ and the corresponding wavelength, oscillator strength *f* and the dipole moment change on excitation Δ*μ* for the monomer, J, V1, V2 and V2′-type ITIC dimers

	Monomer			J-type			V1-type			V2-type			V2′-type		
	*E*_abs_/eV (nm)	*f*	Δ*μ*/D	*E*_abs_/eV (nm)	*f*	Δ*μ*/D	*E*_abs_/eV (nm)	*f*	Δ*μ*/D	*E*_abs_/eV (nm)	*f*	Δ*μ*/D	*E*_abs_/eV (nm)	*f*	Δ*μ*/D
S_1_	1.83 (677)	2.84	0.00	1.73 (717)	4.71	0.00	1.75 (708)	3.22	1.50	1.77 (700)	1.64	3.15	1.76 (704)	0.69	3.26
S_2_	2.19 (566)	0.00	0.00	1.86 (667)	0.00	0.00	1.86 (667)	1.20	0.84	1.80 (689)	1.39	11.80	1.83 (677)	0.50	13.46
S_3_	2.54 (488)	0.20	0.00	1.92 (646)	0.00	0.02	1.92 (646)	0.33	17.12	1.86 (667)	0.47	5.96	1.85 (670)	0.05	20.29
S_4_	2.56 (484)	0.00	0.00	1.98 (626)	1.08	0.02	1.98 (626)	0.89	18.14	1.92 (646)	1.52	10.45	1.88 (659)	3.71	4.54

The proposed mechanism for the intermolecular CT and CD proceeding in the neat ITIC film is illustrated in Scheme 1b. The intermolecular CT from the S_1_ state and CD are triggered by the larger dipole moment change on the excited state after the exciton reaches to the lower symmetric V-type molecular stacking of ITIC. The dissociated holes move to the donor layer, whereas the free electrons remain in the acceptor layer, eventually generate a photocurrent. Direct photoexcitation at the stacking faults with higher energy can more effectively induce intermolecular CT, followed by CD, to form free carriers and holes.

The extent of this CT is governed by the stacking arrangement of π–π conjugation, which could be affected by the side-chain structure as well as the bulkiness around NFAs. Typically, these factors do not directly affect the optical and electronic properties of NFAs. However, this work suggests that the design of peripheral substituents around the core structure of NFAs can modulate stacking faults to increase the dipole moment that pursues the CT and CD in the neat ITIC layer. This new route can minimize the energy offset without the aid of the energy offset of the donor/acceptor heterojunction, and can reduce the energy loss at the junction. This insight will be instrumental in designing new NFA molecules to elevate the PCEs.

## Conclusion

In this study, we have uncovered that intermolecular CT and CD occur within the neat ITIC film even in the absence of a donor layer, although they are believed to proceed at the donor/acceptor interface with the aid of the interfacial energy offset. Femtosecond time-resolved visible to MIR absorption measurements revealed that the intermolecular CT immediately proceeds within 0.1 ps after the photoirradiation, and they are further dissociated into free carriers with a time constant of 56 ps. Theoretical calculations support these experimental results that the stacking faults such as V-type molecular stacking cause a large dipole moment change on the excited state to induce such intermolecular charge separation, whereas J-type stacking does not facilitate such processes. These results demonstrate that a previously unknown pathway will be opened through the distortion of the stacking structures in NFAs. Our study underscores the critical role of controlling the stacking structure of NFAs through molecular design, particularly in tailoring side-chain structures and bulkiness around NFAs. This newfound understanding will be pivotal in guiding the design of novel NFA molecules aimed to minimize voltage loss in OPVs.

## Data availability

The data supporting the findings of this study are available within the article and its ESI.† Additional data that support the findings of this study are available from the corresponding author upon reasonable request.

## Author contributions

A. Y. and H. I. conceived the concept and prepared the manuscript with feedback from the other authors. K. K. performed the time-resolved measurements and analysis. T. Ur., M. H. and H. S. performed the theoretical calculation. S. T., K. M. and Y. K. performed the TREPR measurements and analysis. A. Y., K. K., M. H., T. Um. and H. I. discussed the results. A. Y., K. K. and H. I. wrote and revised the paper with contributions from the other authors.

## Conflicts of interest

There are no conflicts to declare.

## Supplementary Material

SC-OLF-D4SC00917G-s001
