# Supplementary material for: Boosting charge separation in organic photovoltaics: unveiling dipole moment variations in excited non-fullerene acceptor layers
Source: Chem Sci. 2024 Jul 10. Online ahead of print. doi: 10.1039/d4sc00917g (PMC11323316; doi:10.1039/d4sc00917g)
Supplement: SC-OLF-D4SC00917G-s001 [file SC-OLF-D4SC00917G-s001.pdf]

Electronic Supplementary Material (ESI) for Chemical Science.
This journal is © The Royal Society of Chemistry 2024

Supplementary Materials for

Boosting Charge Separation in Organic Photovoltaics: Unveiling Dipole Moment Variations in Excited Non-Fullerene Acceptor Layers

Akira Yamakata,[1] Kosaku Kato,[1] Takumi Urakami,[2] Sota Tsujimura,[3] Kasumi Murayama,[3] Masahiro Higashi [4*] Hirofumi Sato,[2] Yasuhiro Kobori, [3,5,6] Tomokazu Umeyama[7] and Hiroshi Imahori[2,8,9*]*

[1]Graduate School of Natural Science and Technology, Okayama University, 3-1-1, Tsushima-naka, Kita-ku, Okayama 700-8530, Japan

[2]Department of Molecular Engineering, Graduate School of Engineering, Kyoto University, Nishikyo-ku, Kyoto 615-8510, Japan

[3] Department of Chemistry, Graduate school of Science, Kobe University, 1-1 Rokkodai-cho, Nada-ku, Kobe, Hyogo, 657-8501, Japan

[4] Department of Complex Systems Science, Graduate School of Informatics, Nagoya University, Furo-cho, Chikusa-ku, Nagoya 464-8601, Japan

[5] Molecular Photoscience Research Center, Kobe University, 1-1 Rokkodai-cho, Nada-ku, Kobe 657-8501, Japan

[6] CREST, JST, Honcho 4-1-8, Kawaguchi, Saitama 332-0012, Japan.

[7]Department of Applied Chemistry, Graduate School of Engineering, University of Hyogo, 2167 Shosha, Himeji, Hyogo 671-2201, Japan

[8]Institute for Integrated Cell-Material Sciences (WPI-iCeMS), Kyoto University, Sakyo-ku, Kyoto 606-8501, Japan

[9]Institute for Liberal Arts and Sciences (ILAS), Kyoto University, Kyoto 606-8316, Japan

Table of Contents

Experimental Section ······ 3
Figure S1 Chemical structures of ITIC and PBDB-T. ······ 5
Figure S2 Steady-state UV-VIS-NIR and MIR absorption spectra of the sample films. ······ 5
Figure S3 Pump fluence dependence of the transient absorption. ······ 6
Figure S4 Time profile of the transient absorption at 930 nm and 1800 cm-1 for neat ITIC film and PBDB-T:ITIC blend film. ······ 7
Figure S5 Transient absorption of the PBDB-T film. ······ 8
Figure S6 Time-resolved EPR spectrum of the blend film of PBDB-T:ITIC. ······ 9
Table S1 TREPR parameters of the photoinduced CS states of PBDB-T:ITIC blend film. ······ 10
Figure S7 EPR parameters of the g-factor and the hyperfine couplings. ······ 11
Figure S8 Time-resolved EPR spectrum of the neat ITIC film. ······ 15
Figure S9 Calculated acceptor part of ITIC. ······ 16
Table S2 Vibrational frequency of the CN group obtained by the experiment and calculation ······ 16
Figure S10 Structures of the ITIC dimers for the theoretical calculation. ······ 17
Figure S11 Electron and hole distributions on the excitations of ITIC dimers ······ 18
Figure S12 Results of the molecular dynamics simulation of the ITIC film. ······ 19

Experimental Section

The chemical structures of ITIC and PBDB-T (Poly[[4,8-bis[5-(2-ethylhexyl)-2-thienyl]benzo[1,2-b:4,5-b']dithiophene-2,6-diyl]-2,5-thiophenediyl[5,7-bis(2-ethylhexyl)-4,8-dioxo-4H,8H-benzo[1,2-c:4,5-c']dithiophene-1,3-diyl]]) are shown in Figure S1. The sample films for the TA measurement were prepared by drop-casting a chloroform solution of ITIC, PBDB-T and their blend (1:1 by wt.) onto a CaF_2 substrate with the thickness of ~200 nm. The film was set in an IR cell filled with 20 torr N_2 during the measurement. Steady-state absorption spectra in visible-NIR and MIR region were measured by using a UV–vis spectrometer (Jasco, V-670) and a Fourier transform infrared (FT-IR) spectrometer (Bruker, Vertex 80), respectively. The obtained steady-state absorption spectra are shown in Figure S2.

The TA measurements were carried out by the pump-probe method with a Ti:sapphire regenerative amplifier (Spectra Physics, Solstice. 90 fs duration, 1 kHz repetition rate) and optical parametric amplifiers (OPAs; Spectra Physics, TOPAS Prime). The sample was photoexcited by a pulse from the OPA with the fluence of 2.6 $\mu J\ cm^{-2}$ (pulse energy 25 nJ) unless otherwise noted. For visible and NIR measurement, a super-continuum probe was generated by focusing a 1250 nm OPA signal into a 5 mm-thick BaF_2 plate. After the sample, the probe was dispersed with a polychromator and detected with a CMOS (UNISOKU, PK100-CX) and InGaAs (UNISOKU, NIR-PDA256) photodiode array for measurement in the VIS and NIR regions, respectively. For MIR measurement, the probe pulse was generated by the difference frequency generation between the signal and idler from the OPA in an $AgGaS_2$ crystal. The probe pulse transmitted through the sample was dispersed with a polychromator and was detected with a 128-channel linear MCT array detector (Infrared Systems Development, FPAS-0144). To obtain broadband MIR TA spectra, we performed the measurement in four wavelength ranges and then merged these results. In order to clarify the changes in the CN vibrations, the spectrum in Figure 4 was obtained by applying a linear baseline correction over the range of 2088 cm^{-1} to 2289 cm^{-1}, which is wide enough to observe these changes. The global analysis was performed by using the GLOTARAN software.[1]

Time-resolved EPR (TREPR) spectra were measured using a Bruker EMX X-band continuous wave (CW) EPR spectrometer without magnetic field modulation at 80 K. Continuum optical parametric oscillators (OPO) systems (Surelite OPO Plus) pumped with a third harmonics (355 nm) of a Nd:YAG laser (Continuum, Surelite I-10, 5 ns) was used for the laser irradiations of 710 nm pulses. A laser de-polarizer (SIGMA KOKI, DEQ 1N) was placed between the laser exit and the microwave cavity. Temperature was controlled by a cryostat system (Oxford, ESR900) by using liquid nitrogen as the cryogen. The samples for TREPR was prepared as follows: ITIC (>98.5%, Sigma-Aldrich), PBDB-T (>99.0%, Sigma-Aldrich), 1,8-diiodooctane (DIO) (>95.0%, TOKYO CHMICAL

INDUSTRY) and chlorobenzene (>99.0%, FUJIFILM Wako Pure Chemical Corporation) were used as received without further purification. ITIC (4 mg), PBDB-T (4 mg), and DIO (2 μL) were dissolved in chlorobenzene (400 μL) and stirred for half day in a glovebox. The bulk-heterojunction (BHJ) thin films were fabricated by the spin-coating of the solutions on thin cover glasses with 1 cm^2 using a spin coater (Mikasa MS-A100). The films were then wrapped in pharmaceutical wrapping paper and processed manually to produce a number of cut films of approximately 0.5 mm^2, which were placed in EPR sample tubes. The films were then degassed using a vacuum line under a vacuum pressure of 10^{-3} Pa to remove solvent and oxygen from the film. After evaporation, nitrogen gas was introduced into the EPR tube; the sample tube was sealed before the TREPR measurement.

S1 Chemical structures of ITIC and PBDB-T

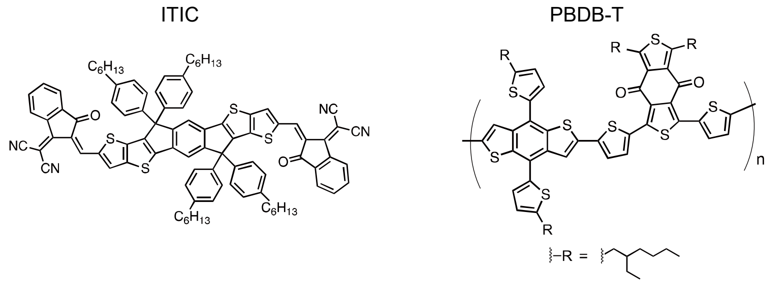

Figure S1 Chemical structures of ITIC and PBDB-T.

S2 Steady state absorption of PBDB-T, ITIC, and their blend film

The steady-state visible-NIR absorption spectra of the PBDB-T, ITIC, and their blend film are shown in Figure S2. The peak of the absorption by PBDB-T and ITIC films exists around 580 nm and 710 nm, respectively, which we adopted as their excitation wavelengths. The FT-IR spectra are shown in Figure S4. The absorption peaks around 2900 cm^{-1} are due to C-H stretching modes. For ITIC and blend film, a sharp peak also exist around 2218 cm^{-1}. This is ascribed to the vibration of CN groups.

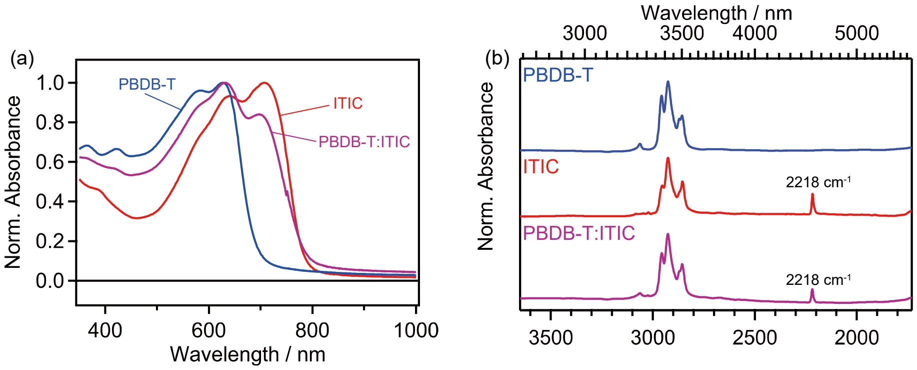

Figure S2 (a) Steady-state UV-VIS-NIR absorption spectra of the ITIC, PBDB-T and PBDB-T:ITIC films. (b) Steady-state FT-IR spectra of the ITIC, PBDB-T and PBDB-T:ITIC films.

S3 Pump fluence dependence of the transient absorption of ITIC film

To examine the observed MIR-TA in more detail, we investigated the fluence dependence of the transient absorption for the ITIC film. The TA signal intensities at 970 nm, 3100 cm^{-1}, and 1800 cm^{-1} averaged over the 0-0.2 ps time range exhibit an almost linear dependence on the excitation fluence (Figure S3a-c). These intensities, when averaged at 100~150 ps, also increased almost linearly up to 3 μJ, but flattened out beyond 3 μJ (Figure S3d-f). These results suggest that the observed MIR-TA signal is triggered by the one-photon absorption process of the ITIC rather than being influenced by thermal effects up to 3 μJ, and that second-order recombination processes proceed beyond 3 μJ.

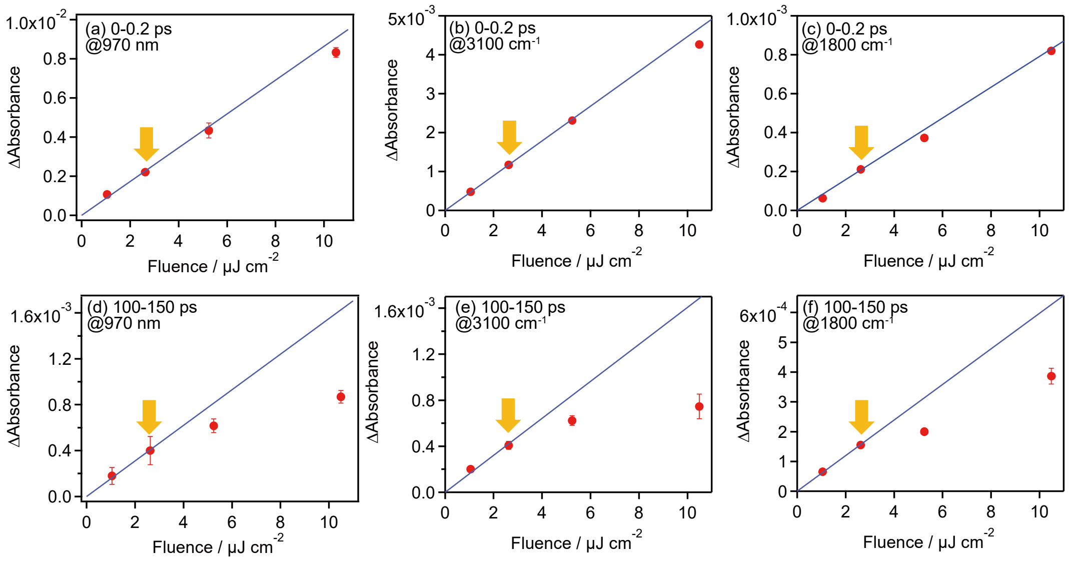

Figure S3 Pump fluence dependence of the transient absorption averaged over 0–0.2 ps and 100–150 ps at 970 nm (a, d), 3100 cm^{-1} (b, e), and 1800 cm^{-1}(c, f), respectively. Arrows indicate the fluence used in this work (2.6 μJ/cm^2). Error bars show the standard deviation of the transient absorption in the time range where the average is taken. Lines are guides to the eye.

S4 Time profile of the transient absorption at 930 nm and 1800 cm^{-1} for neat ITIC film and PBDB-T:ITIC blend films.

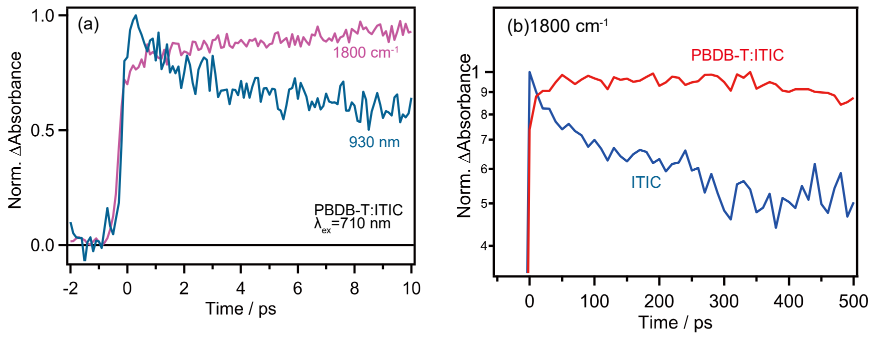

Figure S4 (a) Time profile of the transient absorption at 930 nm and 1800 cm^{-1} for PBDB-T:ITIC blend film. (b) Time profiles of the transient absorption at 1800 cm^{-1} for neat ITIC film and PBDB-T:ITIC blend film.

S5 Transient absorption of PBDB-T film

The visible-NIR TA spectra of the PBDB-T film excited by 580 nm pulses are shown in Figure S5a. A broad positive absorption was observed around 1100 nm, which is originated from the transition of the excited S_1 state to higher states by probe light absorption.[2] The negative absorption around 640 nm is assigned to the ground state bleach of S_0. In the MIR region, another broad absorption peak appeared around 3100 cm^{-1} (~3200 nm, 0.38 eV) as shown in Figure S5b. This peak indicates the existence of excited states with lower binding energy than the S_1 state corresponding to the peak around 1100 nm (~1.1 eV). The time profiles of the TA at 3100 cm^{-1} is compared with those at 640 nm and 1100 nm in Figure S5c. All the TA profiles decayed with similar rate, and almost completely disappeared at 900-1000 ps after excitation (Figure S5b). This result suggests that the excited states observed in the visible-NIR region and that in the MIR region decayed with similar time-constant. PBDB-T was also excited by a 710 nm laser pulse with the same laser fluence (Figure S5d). The transient IR absorption intensity was much smaller than that induced by the 580 nm excitation, but the spectral shape was almost identical. The decay of the intensity at 3100 cm^{-1} was also identical when excited by both 580 nm and 710 nm.

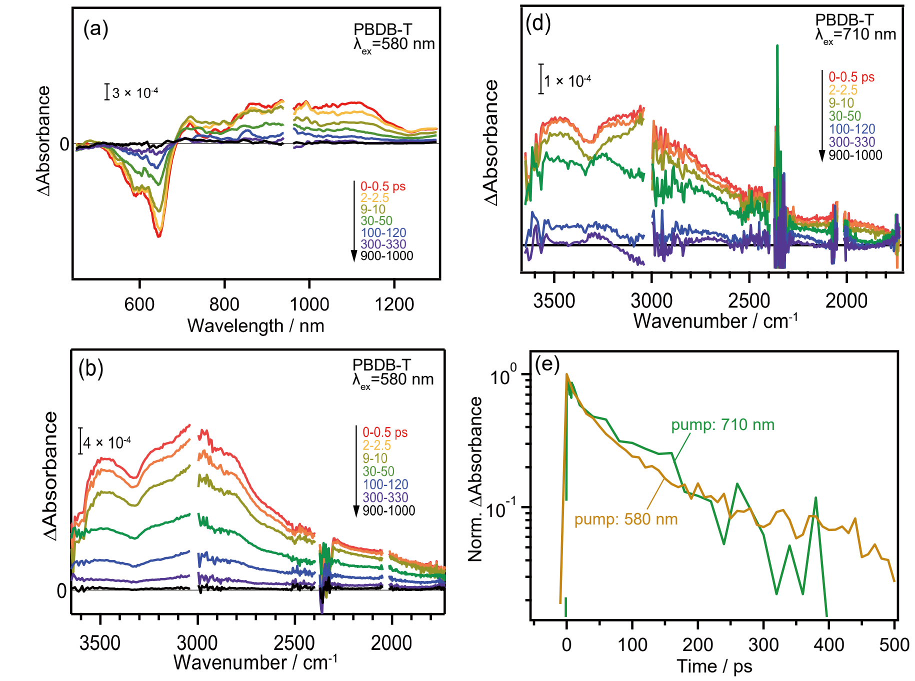

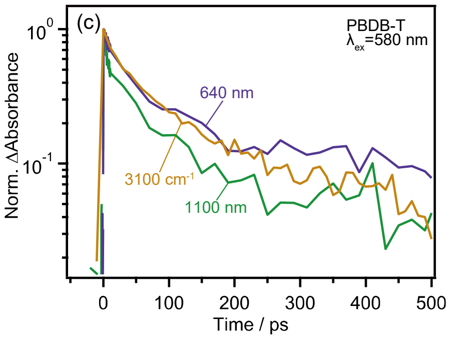

Figure S5 Transient absorption of the PBDB-T film. (a) Visible to NIR region excited by 580 nm. (b) MIR region excited by 580 nm. (c) Time profiles of the TA excited by 580 nm. (d) MIR region excited by 710 nm. (e) Time profiles of the TA at 3100 cm^{-1} excited by 710 nm. The laser fluence was 2.6 μJ cm^{-2}.

S6 Time-resolved EPR measurements of PBDB-T:ITIC blend film

The polaron species formed by photoexcitation of PBDB-T:ITIC blend film was examined by EPR measurements (Figures S6 and S7, Table S1). The emission/absorption (E/A) spin polarization around 343.8 mT corresponds PBDB-$T^{+•}$, while the broad E/A component including the shoulder peak around 343 mT originates from $ITIC^{–•}$ in the spin-correlated radical pair (SCRP).[3] From the red line, the spectrum is explained as a separated polaron pair coupled by the spin-spin dipolar coupling constant (D = -0.26 mT) with J = -0.064 mT as the spin-spin exchange coupling generated by the interfacial charge-separation from the singlet exciton in the ITIC domain of the blend film. The distance between PBDB-$T^{+•}$ and $ITIC^{–•}$ is estimated to be 2.2 nm which is comparable to our reported separation distance (2.1 nm) of the oriented CS states in a PBDB-T:NFA blend system employing a non-fused acceptor molecule.[4] For the neat ITIC film (Figure S8), only a trace of small triplet ITIC signal was observed and the polaron species was not obvious after 300 ns. This discrepancy with the results of fs-TRIR would be ascribed to the difference in the time-resolution of these measurements (hundreds of ns and fs, respectively) where most of charge carriers are recombined at 300 ns since the charge separation efficiency in the neat ITIC is lower than that of the blend film.

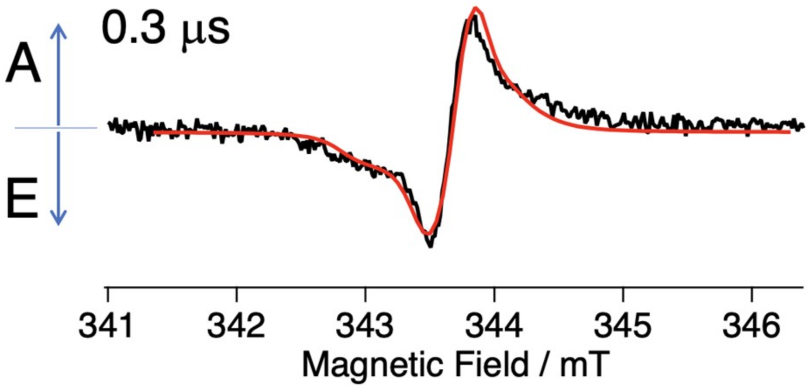

Figure S6. Time-resolved EPR spectrum of the blend film of PBDB-T:ITIC (1 : 1 weight ratio) observed at 80 K after the laser excitation of 710 nm. Detection time is 300 ns after the laser flash. The microwave frequency is 9634.639 MHz. The red line shows simulated spectrum by the spin-correlated radical pair (SCRP) model[3] composed of PBDB-$T^{+•}$ and $ITIC^{–•}$ with a singlet precursor.

Table S1. TREPR parameters of the photoinduced CS states of PBDB-T:ITIC blend film.[a)]

g-tensor (principal values in PBDB-T$^{+\bullet}$) [b)]	g-tensor (principal values in ITIC$^{-\bullet}$) [c)]	Dipolar coupling constant [d)]	Dipolar angles from the g-principal axes in ITIC$^{-\bullet}$	Exchange coupling [d)]
g_z = 2.0021<br>g_y = 2.0028<br>g_x = 2.0033	g_Z = 2.0020<br>g_Y = 2.0034<br>g_X = 2.0046	D = -0.26 (± 0.05) mT	θ_d = 60 (± 10) degrees<br>ϕ_d = 40 (± 10) degrees	J = -0.064 (± 0.01) mT

a) See ref.[3] for the computation method of the EPR spectrum calculation using the SCRP model.

b) Isotropic hyperfine couplings by two equivalent protons (a_{iso} = 0.05 mT) were also considered. Conformation of g-tensor principal axis system are represented by the Euler rotation matrix with x-convention by using α = -20, β = 60 and γ = 50 degrees with respect to the principal axis system in the g-tensor of ITIC$^{-\bullet}$.

c) Anisotropic hyperfine couplings by two equivalent protons (A_X = -0.125 mT, A_Y = -0.426 mT, A_Z = -0.320 mT) and (A_X = -0.071 mT, A_Y = 0.232 mT, A_Z = 0.187 mT) were considered, as shown by the circles in Figure S7. Anisotropic nitrogen hyperfine couplings by four equivalent nuclei (A_{NX} = 0 mT, A_{NY} = 0 mT, A_{NZ} = 0.12 mT) were also considered. The EPR parameters of the g-factor and the hyperfine couplings were estimated by the ORCA 5.0.2 program package for the radical anion. Before this, optimized geometry was obtained by using Gaussian 16 at B3LYP/6- 31G(d,p) level of theory. The other minor hyperfine couplings were treated to be included in the Lorentzian linewidth ($1/T_2 = 1.7 \times 10^7$ s^{-1}).

d) Longitudinal spin relaxation times (T_{23} = 3 μs and T_1 = 3.6 μs) are set to be long enough caused by the modulations in the exchange interaction and in the dipolar interaction.

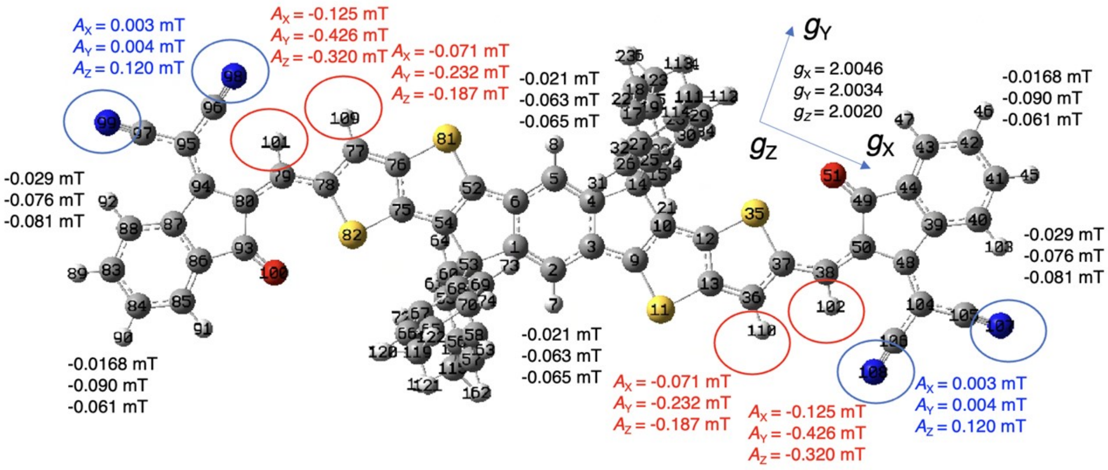

Figure S7. EPR parameters of the g-factor and the hyperfine couplings estimated by the ORCA 5.0.2 program package for the ITIC radical anion. The optimized geometry was obtained by using Gaussian 16 at B3LYP/6- 31G(d,p) level of theory. The minor proton hyperfine parameters are not considered in the present computation of the SCRP model.

Optimized structure of ITIC radical anion represented by Cartesian coordinates (X, Y, Z) of the atoms obtained by using B3LYP/6- 31G(d,p) level of theory.

Atom	X	Y	Z
C	-1.20048584	-0.71957509	0.09965132
C	-0.01701746	-1.43017384	0.09045027
C	1.19362315	-0.70703848	0.09390308
C	1.20056204	0.71953491	0.09962713
C	0.01709259	1.43013214	0.09060306
C	-1.19354662	0.70699483	0.09406572
H	-0.01267686	-2.51558716	0.07379787
H	0.01274238	2.51554707	0.07406992
C	2.55714651	-1.13342850	0.07823375
C	3.43412956	-0.05013685	0.05547192
S	3.35819258	-2.69062171	0.07759783
C	4.77554328	-0.45738298	0.03779847
C	4.92034320	-1.86785126	0.03981869
C	2.64966043	1.26507625	0.08812183
C	3.28996270	1.56560417	-2.37948918
C	2.84153501	2.12476563	-1.17762075
C	2.48305101	3.48079042	-1.18088680
C	2.57292669	4.24466288	-2.34233859

C	3.02818827	3.68858081	-3.54444499
C	3.38157858	2.33532174	-3.53905380
H	3.57015072	0.51832976	-2.41407710
H	2.14081346	3.94881250	-0.26336740
H	2.28620271	5.29334917	-2.31202375
H	3.73750849	1.87121358	-4.45568704
C	3.02281312	2.02549176	1.37999716
C	2.28160594	1.89655701	2.55999060
C	2.68783414	2.52900088	3.73572791
C	3.84720091	3.30960569	3.77984876
C	4.59254877	3.43040185	2.60013135
C	4.18985866	2.80370011	1.42357456
H	1.37811539	1.29728087	2.56740431
H	2.08838521	2.40936823	4.63521262
H	5.50663724	4.01905448	2.59947469
H	4.79077676	2.92705125	0.52856018
S	6.33067449	0.33631630	-0.00804348
C	6.23494637	-2.30402846	0.00534498
C	7.17237059	-1.24373353	-0.02658785
C	8.55111249	-1.51562062	-0.06412012
C	11.96373810	-0.00456713	-0.16422668
C	13.35401564	0.14015613	-0.20344108
C	13.89498502	1.43324693	-0.22021292
C	13.08332168	2.57013756	-0.19896426
C	11.69141803	2.43112163	-0.15970056
C	11.16310292	1.15088481	-0.14310829
H	14.97496837	1.54736685	-0.25064297
H	13.53444830	3.55814257	-0.21303014
H	11.02546339	3.28841156	-0.14238453
C	11.07149837	-1.20021890	-0.13824346
C	9.73385476	0.74843941	-0.10230279
C	9.70414076	-0.72547648	-0.09969924
O	8.78508170	1.52905777	-0.07611890
C	-2.55706713	1.13338729	0.07848271
C	-2.64957925	-1.26511766	0.08820365
C	-3.43406521	0.05010539	0.05564486
C	-3.38079307	-2.33511562	-3.53916717
C	-3.02841802	-3.68869200	-3.54427431
C	-2.57407353	-4.24497714	-2.34197186

C	-2.48408733	-3.48104343	-1.18051412
C	-2.84153201	-2.12479373	-1.17753401
C	-3.28907577	-1.56539010	-2.37967182
H	-3.73597599	-1.87081205	-4.45599770
H	-2.28816001	-5.29387316	-2.31145103
H	-2.14256458	-3.94924356	-0.26281748
H	-3.56845220	-0.51790433	-2.41444909
C	-3.84710426	-3.31020594	3.77962574
C	-4.59232309	-3.43104746	2.59970527
C	-4.18962236	-2.80414645	1.42337779
C	-3.02266183	-2.02561942	1.38004371
C	-2.28160258	-1.89680390	2.56002783
C	-2.68789064	-2.52955333	3.73570107
H	-5.50627020	-4.01994094	2.59881024
H	-4.79030047	-2.92759331	0.52821438
H	-1.37813613	-1.29749962	2.56764253
H	-2.08848754	-2.41010057	4.63522636
C	-4.77547343	0.45738755	0.03797118
C	-4.92024224	1.86786085	0.04008083
C	-6.23482995	2.30408374	0.00559570
C	-7.17228968	1.24382474	-0.02644109
C	-8.55102147	1.51575171	-0.06400316
C	-9.70407128	0.72563866	-0.09967192
S	-3.35807973	2.69059567	0.07794751
S	-6.33064062	-0.33624938	-0.00797183
C	-13.89496581	-1.43297065	-0.22046681
C	-13.08333327	-2.56988325	-0.19924474
C	-11.69142713	-2.43090477	-0.15991632
C	-11.16307990	-1.15068269	-0.14323485
C	-11.96368412	0.00479139	-0.16432491
C	-13.35396351	-0.13989421	-0.20360431
H	-14.97495096	-1.54706077	-0.25094772
H	-13.53448460	-3.55787597	-0.21338192
H	-11.02549540	-3.28821296	-0.14261901
H	-14.01832560	0.71290812	-0.22116342
C	-9.73382316	-0.74827485	-0.10235012
C	-11.07141328	1.20041814	-0.13824291
C	-11.54072142	2.51874846	-0.15165156
C	-10.70519219	3.67365900	-0.13003396

C	-12.92382126	2.86126205	-0.18961897
N	-10.06495048	4.65009087	-0.11388805
N	-14.04318058	3.19184687	-0.22030045
O	-8.78507133	-1.52892075	-0.07615956
H	-8.72445270	2.58583014	-0.06501397
H	8.72457250	-2.58569436	-0.06519204
H	14.01840048	-0.71262809	-0.22101771
C	11.54084006	-2.51853488	-0.15170525
C	12.92395022	-2.86101160	-0.18964609
C	10.70533904	-3.67346800	-0.13017697
N	14.04331848	-3.19156703	-0.22030974
N	10.06512109	-4.64991638	-0.11410568
H	-6.55421234	3.33981759	0.00176503
H	6.55436485	-3.33975097	0.00145750
C	4.27058154	4.01694730	5.04533034
H	5.36024649	4.03406081	5.14769596
H	3.92848637	5.05980480	5.05393284
H	3.85496062	3.53092564	5.93305753
C	-3.16250104	-4.52888617	-4.79206047
H	-4.14139161	-5.02310648	-4.83608534
H	-3.06575305	-3.92016245	-5.69611476
H	-2.40118003	-5.31436926	-4.82971999
C	-4.27138424	-4.01728684	5.04495352
H	-5.36014071	-4.01342163	5.15859966
H	-3.95040827	-5.06683822	5.04325424
H	-3.83713588	-3.54532883	5.93128859
C	3.16192686	4.52891127	-4.79217675
H	4.13643973	5.03214040	-4.83084323
H	3.07602180	3.91856184	-5.69619663
H	2.39368478	5.30740365	-4.83501966

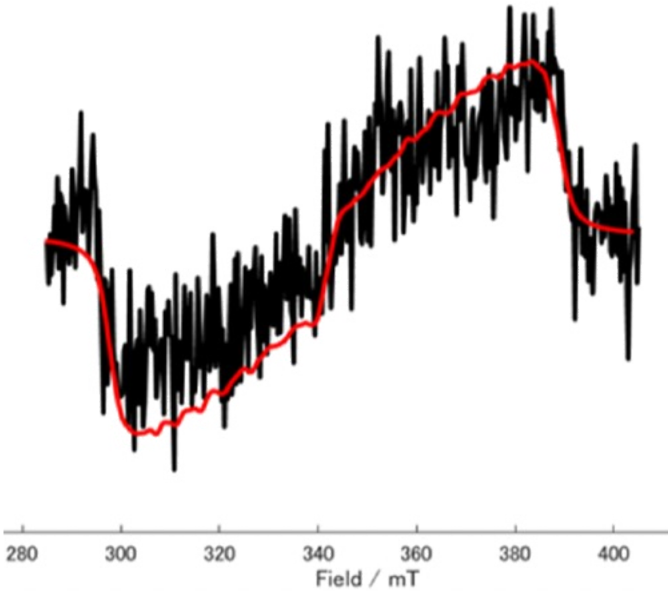

Figure S8. Time-resolved EPR spectrum of the neat film of ITIC observed at 80 K after the laser excitation of 710 nm, showing triplet exciton ^{3}ITIC*. Detection time is 300 ns after the laser flash. The red line is calculated triplet EPR spectrum by using the dipolar coupling constants of D = 46.5 mT and E = 14.0 mT with the anisotropic population ratio in the intersystem crossing of (P_x, P_y, P_z) = (0.45, 0.55, 0).

S7 Theoretical calculation on the vibrational frequency of the CN group

Density functional theory (DFT) and time-dependent DFT (TDDFT) calculations were performed to understand the origin of the frequency shift of the CN group observed in the experiment. The calculations were performed at the B3LYP-D3/6-31+G(d) level using Gaussian 16.[5] To evaluate the localization effect of an electron / hole around the CN group, the calculations were performed for ITIC monomer (where the long alkyl chains were replaced with methyl group) as well as the acceptor part of ITIC capped with methyl group (Figure S9). The vibrational frequency of the CN group was evaluated for the anion and cation as well as S_0 and S_1 of this molecular structure. The calculation results are shown in Table S2. The results show that the vibrational frequency of the CN group is red-shifted (blue-shifted) when the density of electron around the CN group increased (decreased). It is also found that the IR intensities of cations are smaller than those of S_1 and anion, which could be the reason that the frequencies corresponding to the cation have not been observed in the experiment.

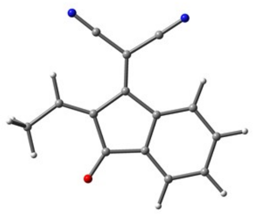

Figure S9. Calculated acceptor part of ITIC.

Table S2 Vibrational frequency (in cm^{-1}) of the CN group obtained by the experiment and DFT calculation. Calculated IR intensities (in km/mol) are also shown in parentheses.

	Molecule	S_0	S_1	Anion	Cation
Experiment	ITIC	2220	2205	2175	---
Calculation	ITIC	2322<br>(492)	2303<br>(17363)	2296<br>(7843)	2330<br>(167)
	acceptor	2331<br>(57)	2297<br>(278)	2261<br>(332)	2372<br>(68)

S8 Theoretical calculation on the photophysical properties of ITIC monomer and 4-type dimers

DFT and TDDFT calculations were performed to investigate the photophysical properties of ITIC monomer and four types of dimers (Figure S10). Geometry optimizations were performed in the ground states at the B3LYP-D3/6-31G(d) level. Then the photophysical properties were investigated with the PCM-tuned ωB97X-D functional[6] and 6-31G(d) basis set. The calculated absorption spectra are shown in Figure 6. The absorption energies, oscillator strengths, and the electric dipole moment changes are summarized in Table 1. The hole-electron analysis was also performed using Multiwfn program[7] (Figure S11). It is found that the hole and electron are evenly delocalized over the J-type dimer whereas the hole and electron are localized in one monomer in the V2 and V2'-type dimers.

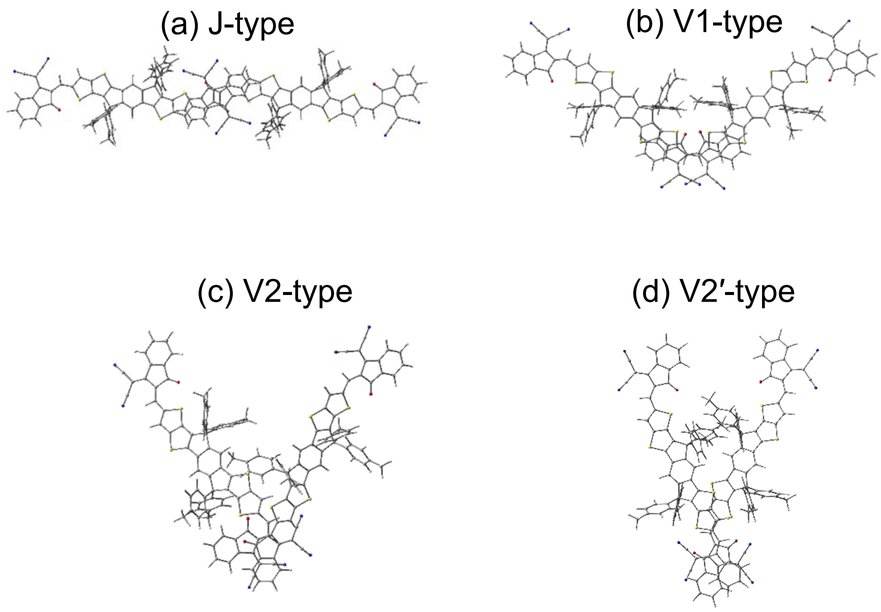

Figure S10. Structures of the four types of the ITIC dimers for the theoretical calculation. (a) J-type, (b) V1-type, (c) V2-type, and (d) V2'-type

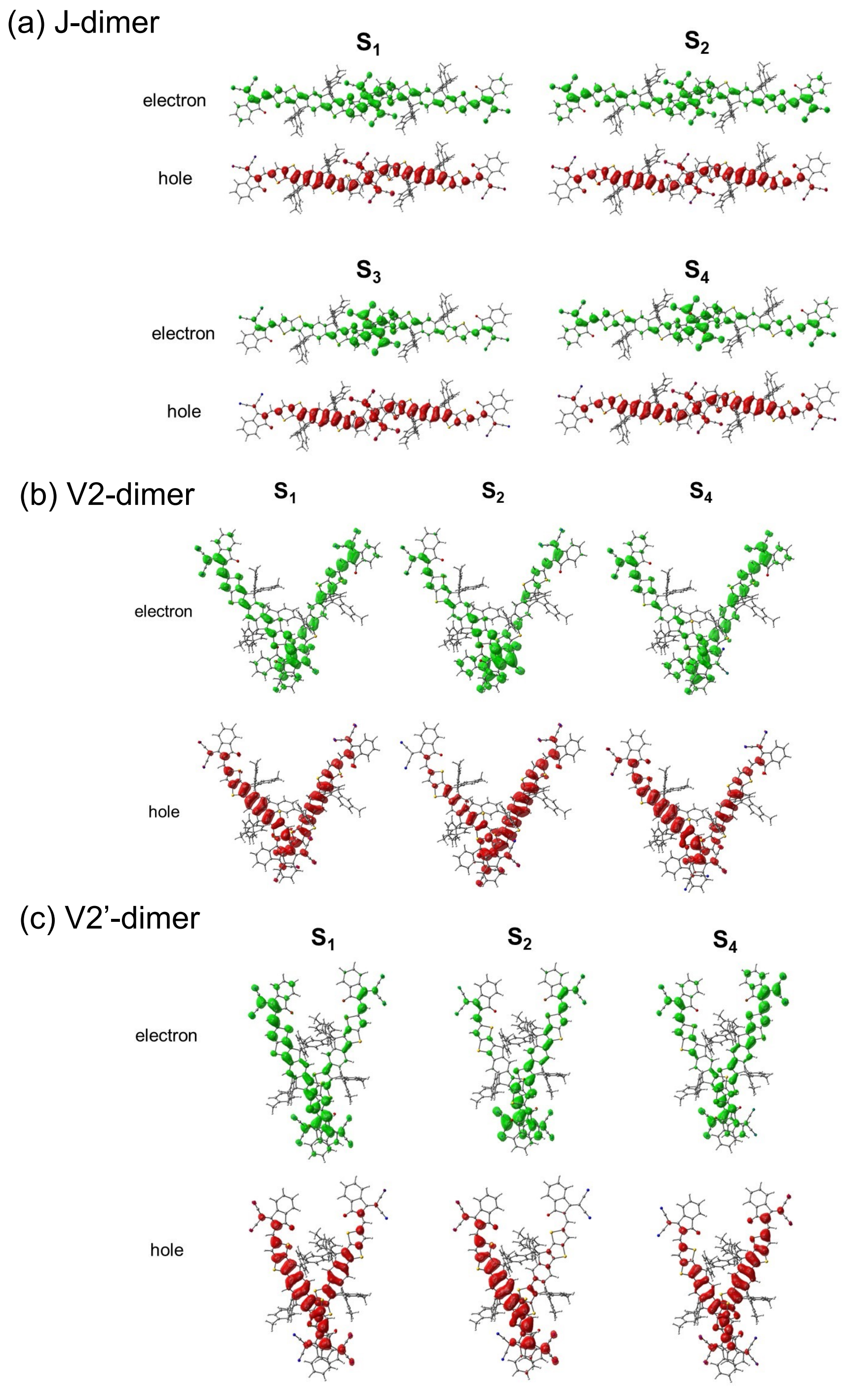

Figure S11. Electron and hole distributions on the excitations of (a) J-dimer, (b) V2-dimer, and (c) V2' dimer of ITIC.

S9 Theoretical analysis of the stacking structures in the ITIC film

To investigate the stacking structures in the ITIC film, molecular dynamics (MD) simulations were performed using the AMBER program package.[8] The general AMBER force field (GAFF) was used. According to the previous study,[9] several steps of NPT-MD simulations were conducted to obtain the film state of ITIC. First, 512 ITIC molecules with full side chains were placed at equal intervals with random orientations in a cubic box with periodic boundary conditions. Next, a 30 ns of NPT-MD simulation was performed at 650 K and 1 atm. Then, the temperature was cooled from 650 K to 300 K at 1 atm in a 100-ns MD simulation. Finally, a 30 ns of NPT-MD simulation was performed at 1 atm and 300 K. The Langevin thermostat and Berendsen barostat were used to control the temperature and pressure. The final structures of 10 independent MD simulations were used for analysis. The calculated radial distribution between acceptor sites of ITIC is shown in Figure S12(a). The peak at ~4 Å indicates the acceptor-acceptor (A-A) stacking, which is consistent with the previous studies. [9,10] The distribution of donor-donor (D-D) distance in the A-A stacking dimer is found to be broad, from ~5 Å to ~28 Å (Figure S12(b)). The peak at ~20 Å corresponds to the J-type dimer whereas the shoulder at ~8 Å corresponds to the V2-type dimer. This result indicates that main dimer structures in the ITIC film is J-type, but that the V2-type structures are also present to some extent. A cluster analysis was also performed as the previous study.[11] Here, the ITIC molecule was described as 13 beads: 1 for the donor site, 6 for the donor-acceptor bridge site, 6 for the acceptor site. Based on the distances between beads of stacking dimers, the principal component analysis (PCA) was performed (Figure S12(c)). It is found that the principal components 1 and 2 approximately correspond to the angle between the two molecules and the position of the interaction, respectively. Then, the data was divided into five clusters by Ward's method (Figure S12(d)). The cluster analysis also showed that the J-type dimer (cluster 0) is dominant whereas the V1- and V2-type dimers (cluster 1-3) are considerably present in the ITIC film.

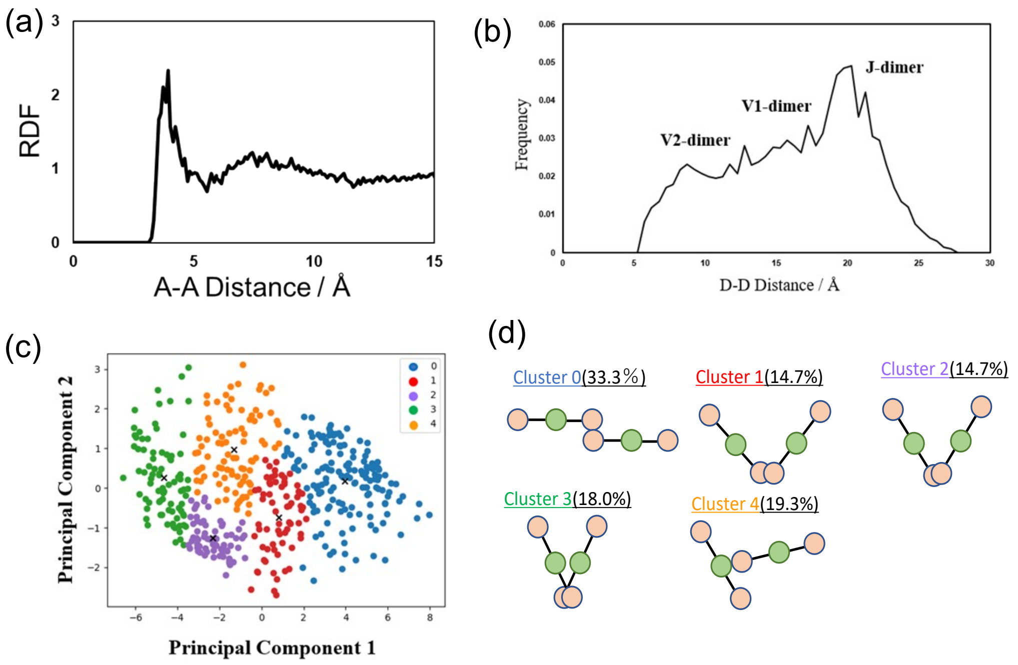

Figure S12. Results of the molecular dynamics simulation of the ITIC film. (a) The radial distribution function between acceptor-acceptor sites. (b) The distribution of donor-donor distance in the acceptor-acceptor stacking dimer. (c) The principal component analysis obtained from cluster analysis. (d) Schematic illustrations of typical dimer structures corresponding to 5 clusters. The green and orange circles represent the donor and acceptor sites, respectively.

References

1 J. J. Snellenburg, S. P. Laptenok, R. Seger, K. M. Mullen and I. H. M. van Stokkum, *J. Stat. Softw.*, 2012, **49**, 1–22.

2 T. R. Hopper, D. Qian, L. Yang, X. Wang, K. Zhou, R. Kumar, W. Ma, C. He, J. Hou, F. Gao and A. A. Bakulin, *Chem. Mater.*, 2019, **31**, 6860–6869.

3 M. Hasegawa, H. Nagashima, R. Minobe, T. Tachikawa, H. Mino and Y. Kobori, *J. Phys. Chem. Lett.*, 2017, **8**, 1179–1184.

4 S. Jinnai, K. Murayama, K. Nagai, M. Mineshita, K. Kato, A. Muraoka, A. Yamakata, A. Saeki, Y. Kobori and Y. Ie, *J. Mater. Chem. A*, 2022, **10**, 20035–20047.

5 M. J. Frisch, G. W. Trucks, H. B. Schlegel, G. E. Scuseria, M. A. Robb, J. R. Cheeseman, G. Scalmani, V. Barone, G. A. Petersson, H. Nakatsuji, X. Li, M. Caricato, a. V. Marenich, J. Bloino, B. G. Janesko, R. Gomperts, B. Mennucci, H. P. Hratchian, J. V. Ortiz, a. F. Izmaylov, J. L. Sonnenberg, Williams, F. Ding, F. Lipparini, F. Egidi, J. Goings, B. Peng, A. Petrone, T. Henderson, D. Ranasinghe, V. G. Zakrzewski, J. Gao, N. Rega, G. Zheng, W. Liang, M. Hada, M. Ehara, K. Toyota, R. Fukuda, J. Hasegawa, M. Ishida, T. Nakajima, Y. Honda, O. Kitao, H. Nakai, T. Vreven, K. Throssell, J. A. Montgomery Jr., J. E. Peralta, F. Ogliaro, M. J. Bearpark, J. J. Heyd, E. N. Brothers, K. N. Kudin, V. N. Staroverov, T. a. Keith, R. Kobayashi, J. Normand, K. Raghavachari, A. P. Rendell, J. C. Burant, S. S. Iyengar, J. Tomasi, M. Cossi, J. M. Millam, M. Klene, C. Adamo, R. Cammi, J. W. Ochterski, R. L. Martin, K. Morokuma, O. Farkas, J. B. Foresman and D. J. Fox, *Gaussian 16, Revision C.01, Gaussian, Inc., Wallingford CT*, 2016.

6 G. Han and Y. Yi, *J. Phys. Chem. Lett.*, 2019, **10**, 2911–2918.

7 T. Lu and F. Chen, *J. Comput. Chem.*, 2012, **33**, 580–592.

8 D. A. Case, R. M. Betz, D. S. Cerutti, T. E. Cheatham III, T. A. Darden, R. E. Duke, T. J. Giese, H. Gohlke, A. W. Goetz, N. Homeyer, S. Izadi, P. Janowski, J. Kaus, A. Kovalenko, T. S. Lee, S. LeGrand, P. Li, C. Lin, T. Luchko, R. Luo, B. Madej, D. Mermelstein, K. M. Merz, G. Monard, H. Nguyen, H. T. Nguyen, I. Omelyan, A. Onufriev, D. R. Roe, A. Roitberg, C. Sagui, C. L. Simmerling, W. M. Botello-Smith, J. Swails, R. C. Walker, J. Wang, R. M. Wolf, X. Wu, L. Xiao and P. A. Kollman, *Amber 2016, University of California, San Francisco*, 2016.

9 G. Kupgan, X. K. Chen and J. L. Brédas, *Mater. Today Adv.*, 2021, **11**, 100154.

10 G. Han, Y. Guo, X. Song, Y. Wang and Y. Yi, *J. Mater. Chem. C*, 2017, **5**, 4852–4857.

11 K. Imamura, T. Yamamoto and H. Sato, *Chem. Phys. Lett.*, 2020, **742**, 137135.
